# APOC3 induces endothelial dysfunction through TNF-α and JAM-1

**DOI:** 10.1186/s12944-016-0326-0

**Published:** 2016-09-13

**Authors:** Yun Tao, Yisong Xiong, Huimin Wang, Shaopeng Chu, Renqian Zhong, Jianxin Wang, Guihua Wang, Xiumei Ren, Juan Yu

**Affiliations:** 1 Center of Laboratory Medicine, Affiliated Hospital, Nantong University, 20 Xi Si Road, Nantong, 226001 People’s Republic of China; 2 Department of Laboratory Medicine, Chengdu Military General Hospital, 270 Tian Hui Road, Chengdu, 610000 People’s Republic of China; 3 Department of Laboratory Medicine, Changzheng Hospital, Second Military Medical University, 415 Feng Yang Road, Shanghai, 200003 People’s Republic of China; 4 Institute of Public Health, Nantong University, 9 Se Yuan Road, Nantong, 226001 People’s Republic of China

**Keywords:** APOC3, Inflammation, Cardiovascular disease, Endothelial dysfunction

## Abstract

**Background:**

The fatality rate for cardiovascular disease (CVD) has increased in recent years and higher levels of triglyceride have been shown to be an independent risk factor for atherosclerotic CVD. Dysfunction of endothelial cells (ECs) is also a key factor of CVD. APOC3 is an important molecule in lipid metabolism that is closely associated with hyperlipidemia and an increased risk of developing CVD. But the direct effects of APOC3 on ECs were still unknown. This study was aimed at determining the effects of APOC3 on inflammation, chemotaxis and exudation in ECs.

**Methods:**

ELISA, qRT-PCR, immunofluorescence, flow cytometry and transwell assays were used to investigate the effects of APOC3 on human umbilical vein endothelial cells (HUVECs). SiRNA-induced TNF-α and JAM-1 silencing were used to observe how APOC3 influenced the inflammatory process in the ECs.

**Results:**

Our results showed that APOC3 was closely associated with the inflammatory process in ECs, and that this process was characterized by the increased expression of TNF-α. Inflammatory processes further disrupted the tight junctions (TJs) between HUVECs by causing increased expression of JAM-1. JAM-1 was involved in maintaining the integrity of TJs, and it promoted the assembly of platelets and the exudation of leukocytes. Changes in its expression promoted chemotaxis and the exudation of ECs, which contributed to atherosclerosis. While the integrity of the TJs was disrupted, the adhesion of THP-1 cells to HUVECs was also increased by APOC3.

**Conclusions:**

In this study, we describe the mechanism by which APOC3 causes inflammation, chemotaxis and the exudation of ECs, and we suggest that controlling the inflammatory reactions that are caused by APOC3 may be a new method to treat CVD.

## Background

The fatality rate for CVD has increased in recent years, and it threatens the lives of people around the world. CVD is the basis of coronary heart disease (CHD), higher levels of triglyceride have been shown to be an independent risk factor for atherosclerotic CVD [1]. While all factors related to the concentration of circulating triglycerides are important, APOC3 is of particular concern [2, 3].

APOC3 is a 79-amino acid glycoprotein with a molecular mass of 8.8 kDa that is synthesized mainly in the liver and rarely in the intestines. APOC3 is a component of chylomicrons (CMs), very low density lipoproteins (VLDLs), high-density lipoproteins (HDLs) and low density lipoproteins (LDLs) [4]. It becomes mature in the endoplasmic reticulum [5]. Because it is a crucial molecule in lipid metabolism, the main effect of APOC3 is thought to be its inhibition of the function of lipoprotein lipase (LPLs) by displacing the enzymes from TG-rich particles [6–8]. This process delays lipid metabolism and causes lipid deposits.

It has been reported that there are clear differences in plasma concentrations of APOC3 between different people. Individuals with a higher plasma level of APOC3 are more likely to suffer from CVDs [9–11]. Insulin response element (IRE) is located in the promoter of the APOC3 gene, and it has been shown that APOC3 induces diabetes and insulin resistance [10, 12].

Studies have revealed a strong link between inflammation and abnormal lipid metabolism. APOC3 mediates the metabolism of lipids, which usually co-exists with inflammation. As previously reported, endothelial dysfunction lies at the root of AS, and inflammation occurs throughout the process of AS [13], which leads to more serious diseases. Inflammation, chemotaxis and the exudation of ECs are indispensable in CVD. The integrity of tight junctions (TJs) is also vital. Endothelial permeability is regulated by TJs, and alterations in the expression of the proteins that form these junctions might lead to a leaky endothelial barrier [14–16]. Disruptions in TJs affect lipid deposition, which causes CVD.

However, the mechanism by which APOC3 leads to CVD and whether this process is associated with the destruction of the functions of cardiovascular ECs and inflammation remain unknown. This study was aimed at determining the effects of APOC3 on inflammation, chemotaxis and exudation in ECs.

## Methods

### Materials

HUVECs and THP-1 monocytes were obtained from Science Cell Lab (USA). APOC3 was acquired from ACADEMY Corporation (USA). Dulbecco’s modified Eagle medium (DMEM), foetal bovine serum (FBS) and trypsin were obtained from Science Cell Lab. The TNF-α ELISA assay kit was obtained from Youersheng Corporation (China). The RNA extraction reagent was purchased from Generay Corporation. SuperReal PreMix Plus was obtained from Tiangen Corporation. The RT-reaction Kit was purchased from Fermentas Corporation. PCR primers were purchased from Shenggong Corporation (China). All siRNAs were obtained from JIMA Corporation (China). All of the antibodies used in this study were purchased from Santa Cruz Biotechnology and Alexa. Transwell chambers were purchased from Fisher Scientific Corporation.

### Methods

#### Cell culture and treatment

HUVECs were cultured in DMEM supplemented with 10 % foetal bovine serum. After the cells reached confluence, they were washed twice with PBS and isolated at 37 °C using a trypsin solution.

To test the effect of APOC3 on HUVECs, APOC3 was added to the culture medium after 72 h in culture. After 48 h in culture and before the cells were treated with APOC3, some of the cells were transfected with TNF-α siRNA or JAM-1 siRNA.

To test the effect of exogenous TNF-α on HUVECs, TNF-α was added to the culture medium after 72 h in culture. After 2,4 or 16 h in culture, the secretion of JAM-1 were tested by qRT-PCR.

#### Transfection with TNF-α siRNA or JAM-1 siRNA

Transfection with TNF-α siRNA or JAM-1 siRNA was performed using Lipofectamine 2000 (Life Technologies) according to the manufacturer’s instructions. The siRNA-lipid complexes were added to the cells, and the medium was replaced 6 h later. QRT-PCR was performed 24 h after the cells were transfected. To confirm whether the cells had been successfully transfected, an inverse fluorescence microscope was used to confirm that the fluorescent signal of the siRNA was present in the cells. The sequences of the siRNAs were as follows:

- TNF-α Forward: GCCUGUAGCCCAUGUUGUATT
- TNF-α Reverse: UACAACAUGGGCUACAGGCTT
- JAM-1 Forward: GUCGAGAGGAAACUGUUGUTT
- JAM-1 Reverse: ACAACAGUUUCCUCUCGACTT

#### ELISA

The concentration of TNF-α was tested according to the manufacturer’s instructions. All of the samples that contained different concentrations of TNF-α were placed at 37 °C for 2 h. We then added working solution A and incubated the samples at 37 °C for 1 h. After the plates were washed, we added working solution B to the samples and incubated them at 37 °C for 30 min. The plates were washed again. Next, the substrate solution was added to the samples, and the cells were incubated at 37 °C for 20 min. Finally, stop buffer was added to the samples, and the absorbance was read at 450 nm.

#### RNA purification and qRT-PCR

Total RNA was purified from the treated cells using Trizol reagent according to the manufacturer’s instructions. The purity and quantity of the RNA were measured using a spectrophotometer by testing its optical density at 260 nm and 280 nm, and the quality of the RNA was determined using agarose gel electrophoresis. Then the RNA was subjected to reverse transcription with a RT-reaction Kit. CDNA was amplified from the purified RNA and then quantified using a CFX connect Real-Time PCR System in 20-μl reaction volumes with SuperReal PreMix Plus. The reaction volumes contained 8 μl of diluted cDNA template, 1 μl each of the forward and reverse primers (10 μM) and 10 μl of 2 × SuperReal PreMix Plus. All of the reactions were performed in triplicate. The relative quantities of our target mRNAs were normalized to the level of GAPDH. The following primers were used:

- Homo GAPDH Forward: AGAAGGCTGGGGCTCATTTG
- Homo GAPDH Reverse: AGGGGCCATCCACAGTCTTC
- Homo TNFα Forward: 5‘CCGAGTGACAAGCCTGTAGCC 3’
- Homo TNFα Reverse: 5‘TTGAAGAGGACCTGGGAGTAGATG 3’
- Homo JAM-1 Forward: 5‘CACGGAATGGGTATGGGACAC 3’
- Homo JAM-1 Reverse: 5‘CCAGGAGAATCAGGGTTACAAGGAC 3’

#### Flow cytometry

After the cells were washed and fixed, the HUVECs were treated with TNF-α antibody for one night at 4 °C. They were then incubated with a secondary antibody to analyse the expression of cell surface TNFα or JAM-1 using an Accuri C6 Flow Cytometer (BD Biosciences).

#### Immunofluorescence

After the cells were washed and fixed, the HUVECs were treated with JAM-1 antibodies overnight at 4 °C. They were then incubated with separate secondary antibodies. The cells were incubated at room temperature in the dark for 1 h. Next, the cells were washed with PBS, and a DAPI solution was added to them. Then the cells were incubated at room temperature in the dark for 20 min. After the cells were washed, a fluorescence microscope was used to observe the expression of JAM-1.

#### Transwell test

HUVECs of the same cell density were cultured in the lower chambers of Transwell chambers for 2 h prior to the experiment. Then THP-1 cells were inoculated into the upper chamber of the Transwell chambers. After the cells were co-cultured for 16 h, the number of THP-1 cells that had migrated to the lower chamber were counted.

#### Statistical analysis

All of the data in these experiments are reported as the mean ± SD. One-way ANOVA was used to analyse all of the data. Data with p-values that were less than 0.05 were considered significant.

## Results

### APOC3 stimulates the secretion of TNF-α in HUVECs

**Fig. 1:**
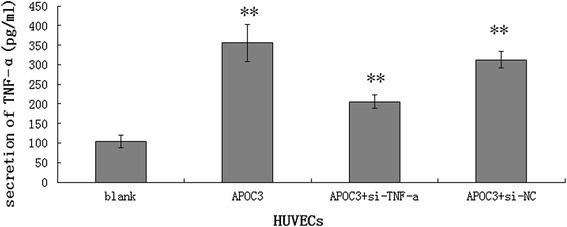
Effects of APOC3 on the secretion of TNF-α in HUVECs. Blank: Untreated HUVECS; APOC3: HUVECs that were incubated with APOC3 (100 μg/ml) for 24 h; APOC3 + siTNF-α: HUVECs that were transfected with TNF-α siRNA before they were incubated with APOC3 (100 μg/ml) for 24 h; APOC3 + siNC: HUVECs that were transfected with blank siRNA before they were incubated with APOC3 (100 μg/ml) for 24 h. Each bar represents the mean ± SE of 3 independent experiments. ** *P* < 0.01 indicates a significant difference compared to the untreated HUVECs

To investigate the role of APOC3 in cell inflammation, ELISA tests were used to determine whether APOC3 influences the secretion of TNF-α in HUVECs. When cells were treated with APOC3, they secreted more TNF-α (355.3 ± 47.114 pg/ml) than the untreated controls (103.8 ± 16.620 pg/ml). However, when TNF-α siRNA was transfected into cells before they were treated with APOC3, the secretion of TNF-α was clearly lower (205.6 ± 17.332 pg/ml) (*P* < 0.01) (Fig. 1). In summary, APOC3 is closely associated with inflammation in ECs, and inflammatory processes are one of the causes of AS.

### APOC3 disrupts TJs partly via TNF-α

**Fig. 2:**
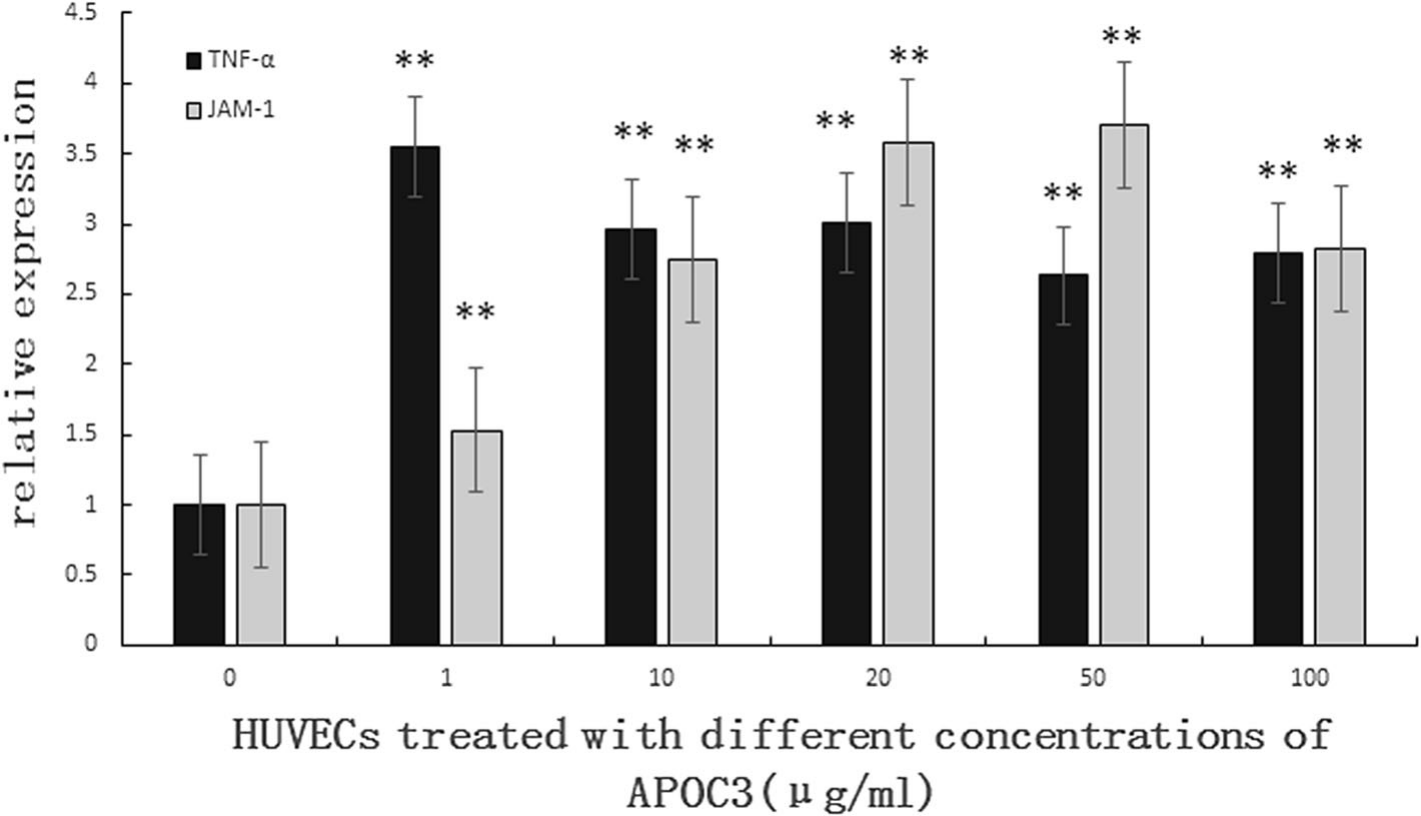
Effect of different concentrations of APOC3 on the expression of TNF-α, JAM-1 in HUVECs according to qRT-PCR. 0: Untreated HUVECS; 1: HUVECs that were incubated with APOC3 (1 μg/ml) for 24 h; 10: HUVECs that were incubated with APOC3 (10 μg/ml) for 24 h;.20: HUVECs that were incubated with APOC3 (20 μg/ml) for 24 h; 50: HUVECs that were incubated with APOC3 (50 μg/ml) for 24 h; 100: HUVECs that were incubated with APOC3 (100 μg/ml) for 24 h. Each bar represents the mean ± SE of 3 independent experiments. ** *P* < 0.01, significantly different from untreated HUVECs

Consistent with the results of ELISA, qRT-PCR showed that APOC3 up-regulated the expression of TNF-α. The expression level of JAM-1, which is an important molecule in the TJ barrier, was also increased. The expression of JAM-1 increased with the increasing concentration of APOC3 (Fig. 2).

**Fig. 3:**
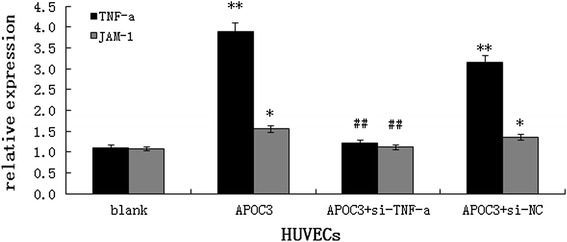
Effect of APOC3 on the mRNA expression levels of TNF-α, JAM-1 in HUVECs according to qRT-PCR. Blank: Untreated HUVECS; APOC3: HUVECs that were incubated with APOC3 (100 μg/ml) for 24 h; APOC3 + siTNF-α: HUVECs that were transfected with TNF-α siRNA before they were incubated with APOC3 (100 μg/ml) for 24 h; APOC3 + siNC: HUVECs that were transfected with blank siRNA before they were incubated with APOC3 (100 μg/ml) for 24 h. Each bar represents the mean ± SE of 3 independent experiments. ** *P* < 0.01, significantly different from untreated HUVECs; * *P* < 0.05, significantly different from untreated HUVECs; ## *P* < 0.01, significantly different from HUVECs incubated with APOC3

To investigate the effect of APOC3 on the expression of JAM-1, we transfected TNF-α siRNA into HUVECs. As the results of qRT-PCR showed, when cells were transfected with the TNF-α siRNA, TNF-α expression was attenuated (Fig. 3). Based on these data, cells were treated with APOC3 and incubated for 24 h. Then, qRT-PCR, flow cytometry (FCM) and immunofluorescence techniques were used to analyse the expression of JAM-1.

**Fig. 4:**
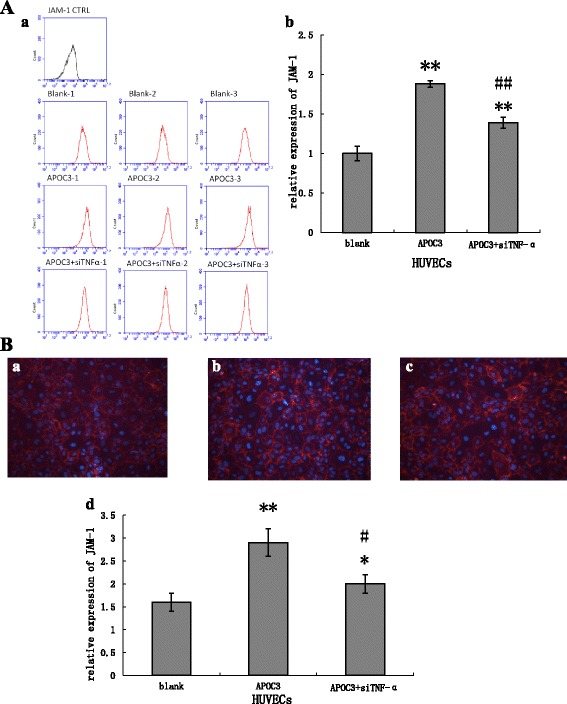
The expression of JAM-1 in HUVECs in protein level. **A** FCM results. Blank: Untreated HUVECS; APOC3: HUVECs that were incubated with APOC3 (100 μg/ml) for 24 h; APOC3 + siTNF-α: HUVECs that were transfected with TNF-α siRNA before they were incubated with APOC3 (100 μg/ml) for 24 h. Each bar represents the mean ± SE of 3 independent experiments. ** *P* < 0.01, significantly different from untreated HUVECs; ## *P* < 0.01, significantly different from HUVECs that were incubated with APOC3. **B** immunofluorescence results. **a** Untreated HUVECS; **b** HUVECs that were incubated with APOC3 (100 μg/ml) for 24 h; **c** HUVECs that were transfected with TNF-α siRNA before they were incubated with APOC3 (100 μg/ml) for 24 h; **d** relative expression levels of JAM-1 in HUVECS. Blank: Untreated HUVECS; APOC3: HUVECs that were incubated with APOC3 (100 μg/ml) for 24 h; APOC3 + siTNF-α: HUVECs that were transfected with TNF-α siRNA before they were incubated with APOC3 (100 μg/ml) for 24 h. Each bar represents the mean ± SE of 3 independent experiments. ** *P* < 0.01, significantly different from untreated HUVECs; * *P* < 0.05, significantly different from untreated HUVECs; # *P* < 0.05, significantly different from HUVECs that were incubated with APOC3

Our results showed that APOC3 up-regulated JAM-1 expression at both the mRNA and protein levels, about double the expression of untreated controls. When HUVECs were transfected with TNF-α siRNA, before treated with APOC3, the expression of JAM-1 was reduced. During this process, the expression of JAM-1 failed to return to baseline level, showing that APOC3 disrupted TJs partly via TNF-α. (Figs. 3 and 4).

**Fig. 5:**
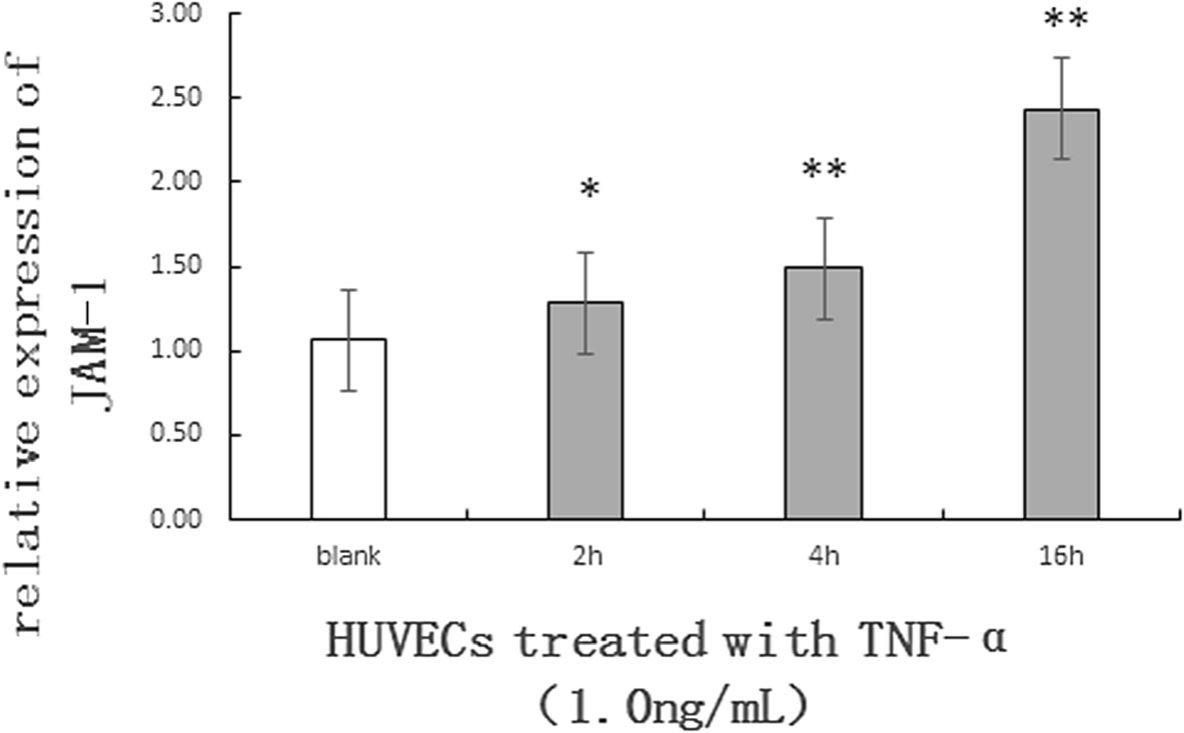
The expression of JAM-1 in HUVECs according to qRT-PCR. Blank: Untreated HUVECS; 2 h: HUVECs that were incubated with TNF-α (1.0 mg/ml) for 2 h; 4 h: HUVECs that were incubated with TNF-α (1.0 mg/ml) for 4 h; 16 h: HUVECs that were incubated with TNF-α (1.0 mg/ml) for 16 h. Each bar represents the mean ± SE of 3 independent experiments. ** *P* < 0.01, significantly different from untreated HUVECs; *P < 0.05, significantly different from untreated HUVECs

Considering the effect of exogenous TNF-α on the expression of JAM-1, the HUVECs were also treated with TNF-α. When cells were treated with TNF-α, the secretion of JAM-1 were up-regulated. When the cells were treated with TNF-αfor 16 h,the secretion of JAM-1 increased to approximately 2.43 times than normal cells (Fig. 5).

### APOC3 promotes the adhesion of THP-1 cells to HUVECs via TNF-α and JAM-1

**Fig. 6:**
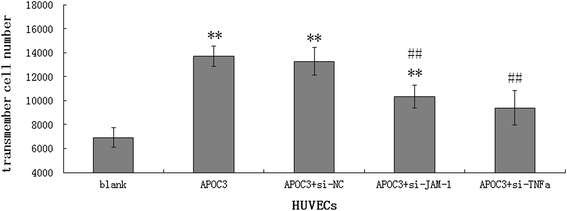
The adhesion of THP-1 cells to HUVECs. Blank: Untreated HUVECS; APOC3: HUVECs that were incubated with APOC3 (100 μg/ml) for 16 h; APOC3 + siNC: HUVECs that were transfected with a negative control siRNA before they were incubated with APOC3 (100 μg/ml) for 16 h; APOC3 + siJAM-1: HUVECs that were transfected with JAM-1 siRNA before they were incubated with APOC3 (100 μg/ml) for 16 h.; APOC3 + siTNF-α: HUVECs that were transfected with TNF-α siRNA before they were incubated with APOC3 (100 μg/ml) for 16 h. HUVECS in each group were in the same cell density. Each bar represents the mean ± SE of 3 independent experiments. ** *P* < 0.01, significantly different from untreated HUVECs; ## *P* < 0.01, significantly different from HUVECs that were incubated with APOC3

THP-1 cells are monocytes. In this study, THP-1 cells were used to investigate the effect of APOC3 on the adhesion of monocytes to ECs. Transwell chamber assays were used to determine the extent of adhesion. After 16 h in co-culture, the number of THP-1 cells that had adhered to the HUVECs was counted. When APOC3 was added to the HUVEC culture medium, the number of transmembrane THP-1 cells clearly increased to approximately 1.98-fold the number that was observed in wells that were grown without adding APOC3. When TNF-α siRNA or JAM-1 siRNA were transfected into the HUVECs before APOC3 was added to the HUVEC culture medium, cell adhesion was clearly reduced. However, the adhesion of THP-1 cells to HUVECS remained significantly higher in the cells that were transfected with JAM-1 siRNA than in the untreated cells, indicating that the effect of APOC3 on adhesion in THP-1 cells was not completely inhibited when we interfered with the expression of JAM-1, indicating that other molecules are likely to affect this process. These results were consistent with our previous results. Nevertheless, the number of migrated THP-1 cells was statistically lower in the cells treated with siRNA and APOC3 than in those treated with APOC3 alone, indicating that the siRNA had an inhibitory effect on APOC3 (Fig. 6).

## Discussion

Studies have shown that APOC3 concentrations are positively correlated with lipid levels, and APOC3 has been found to be an independent risk factor for CVD [17–19]. APOC3 modulates lipid levels in various ways. Previous data have shown that APOC3 has adverse effects in lipid metabolism [5, 20, 21]. It has also been reported that APOC3 concentrations are associated with CVDs, including hyperlipidaemia, CHD [9], and non-alcoholic fatty liver disease [22]. It has also been proposed that polymorphisms of APOC3 are associated with plasma lipid levels [23–28]. Loss-of function mutations, include R19X, IVS2 + 1G → A, IVS3 + 1G → T and A43T, also contribute to lipid metabolism. We propose that these polymorphisms are associated with cell inflammatory processes [29–35].

Atherosclerosis is an inflammatory disease [13]. Abnormal blood lipid levels can further induce inflammation. Studies have suggested that atherosclerotic lesions are characterized by a series of cellular and molecular inflammatory responses. Endothelial dysfunction has been proposed to be an important factor in this inflammatory process. In this study, we examined the mechanisms by which APOC3 might cause endothelial dysfunctions and cellular inflammation.

TNF-α is one of the most important molecules in cellular inflammation, and it has been demonstrated to have a substantial effect on EC dysfunction. CHD patients have higher plasma TNF-α levels than healthy people, patients who suffer from hyperlipidemia also have high serum concentrations of TNF-α, and TNF-α concentrations are positively associated with VLDL-C concentrations and negatively associated with HDL-C concentrations. TNF-α regulates the expression of NOS and thereby influences the production of NO, which is associated with prediabetic metabolic syndrome [36].

In our study, we found that APOC3 caused inflammation in ECs via TNF-α. The increasing concentration of TNF-α led to an increase in reactive oxygen species that caused endothelial dysfunction. We showed that the inflammation that was caused in these cells disrupted the TJs between ECs by demonstrating the presence of alterations in the expression of JAM-1. These processes resulted in EC dysfunction. Emanuela Mazzon et al. showed that TNF-α had an effect on epithelial cells, caused inflammation, and contributed to TJ permeability [37, 38]. Our results show that TNF-α also affects TJs in ECs.

JAM-1 is localized in and is an important component of the TJs in ECs. It mainly influences cell-cell adhesion. Moreover. JAM-1 was associated with the process of exudation, which is significant in AS. JAM-1 is also located on the surface of platelets and leukocytes. It promotes not only cell-cell adhesion but also the assembly of platelets and the exudation of leukocytes, and interactions between ECs and platelets promote the formation of AS. During the process of the adhesion of platelets to ECs, the N-terminus and the 1st Ig fold domain of JAM-1 play leading roles [39, 40]. It has also been reported that APOC3 plays an important role in neutrophil-mediated responses during inflammation [41]. As our results show, TNF-α overexpression increased expression of JAM-1, which promoted the chemotaxis and exudation of cells to cause AS. Akio Kawakami et al. reported that APOC3 promoted the adhesion of THP-1 cells to HUVECs through PKC-α and NF-kB [17]. In our study, we illustrated that the inflammatory reaction caused by APOC3 also played a key role in this process.

The adhesion of circulating monocytes to ECs plays a crucial role in atherogenesis and is associated with inflammatory processes in ECs. Integrins and other adhesion molecules have been reported to participate in this process [5, 42]. When TJs are disrupted, interactions occur between cells. APOC3 has been shown to induce the expression of PCPLC in THP-1 cells [17]. PKCα was also activated in these cells, and it promoted the adhesion of monocytes to ECs. We found that disruptions in the TJs of ECs were also observed in this process. However, the exact mechanisms that contribute to these processes remain to be investigated.

## Conclusions

With previous studies suggested that APOC3 was closely with the development of hyperlipidemia and CVD. In our study, we demonstrated that APOC3 is closely related to the inflammatory process in cells. It further disrupted TJs between ECs, and eventually caused the dysfunction of ECs. This process is required during the generation of AS. Controlling inflammatory reactions caused by APOC3 in these patients is a promising approach to treating CVD.

## Abbreviations

CHD: Coronary heart disease
CM: Chylomicron
CVD: Cardiovascular disease
EC: Endothelial cell
HDL: High-density lipoprotein
HUVEC: Human umbilical vein endothelial cell
IRE: Insulin response element
JAM: Junctional adhesion molecule
LDL: Low density lipoprotein
NAFLD: Nonalcoholic fatty liver disease
TAMPs: TJ associated Marvel domain proteins
TJ: Tight junction
VLDL: Very low density lipoprotein
